# Subsequent malaria enhances virus-specific T cell immunity in SIV-infected Chinese rhesus macaques

**DOI:** 10.1186/s12964-022-00910-7

**Published:** 2022-07-01

**Authors:** Guangjie Liu, Li Qin, Youjia Li, Siting Zhao, Mikhail Shugay, Yongxiang Yan, Yijian Ye, Yue Chen, Cuizhu Huang, Nashun Bayaer, Dickson Adah, Hui Zhang, Zhong Su, Xiaoping Chen

**Affiliations:** 1 Laboratory of Pathogen Biology, State Key Laboratory of Respiratory Disease, Center for Infection and Immunity, Guangzhou Institutes of Biomedicine and Health, Chinese Academy of Sciences, Guangzhou, China; 2 Laboratory of Immunobiology, State Key Laboratory of Respiratory Disease, Center for Infection and Immunity, Guangzhou Institutes of Biomedicine and Health, Chinese Academy of Sciences, Guangzhou, China; 3 Graduate School, University of Chinese Academy of Sciences, Chinese Academy of Sciences, Beijing, China; 4 The Fist Affiliated Hospital of Shenzhen University, Shenzhen, China; 5 Integrated Chinese and Western Medicine Postdoctoral Research Station, School of Traditional Chinese Medicine, Jinan University, Guangzhou, China; 6 Shenzhen Institute of Geriatrics, Shenzhen, China; 7 Genomics of Adaptive Immunity Laboratory, Shemyakin and Ovchinnikov Institute of Bioorganic Chemistry, Russian Academy of Sciences (RAS), Moscow, Russia; 8 Institute of Human Virology, Zhongshan School of Medicine, Sun Yat-Sen University, Guangzhou, China; 9 CAS Lamvac Biotech Co., Ltd, Guangzhou, China

**Keywords:** *Plasmodium*, SIV, Coinfection, Monkey model, Virus-specific immunity

## Abstract

**Background:**

Coinfection with HIV and *Plasmodium* parasites is fairly common, but the sequence of infection with these two pathogens and their impact on disease progression are poorly understood.

**Methods:**

A Chinese rhesus macaque HIV and *Plasmodium* coinfection model was established to compare the impact of pre-existing and subsequent malaria on the progression of SIV infection.

**Results:**

We found that a pre-existing malaria caused animals to produce a greater number of CD4^+^CCR5^+^ T cells for SIV replication, resulting in higher viral loads. Conversely, subsequent malaria induced a substantially larger proportion of CD4^+^CD28^high^CD95^high^ central memory T cells and a stronger SIV-specific T cell response, maintained the repertoire diversity of SIV-specific T cell receptors, and generated new SIV-specific T cell clonotypes to trace SIV antigenic variation, resulting in improved survival of SIV-infected animals.

**Conclusion:**

The complex outcomes of this study may have important implications for research on human HIV and malaria coinfection. The infection order of the two pathogens (HIV and malaria parasites) should be emphasized.

**Video abstract**

**Supplementary Information:**

The online version contains supplementary material available at 10.1186/s12964-022-00910-7.

## Background

Human immunodeficiency virus (HIV) infection or acquired immunodeficiency syndrome (AIDS) and malaria are highly prevalent and cause severe health problems worldwide. At the end of 2019, more than 38 million people were estimated to be living with HIV, resulting in 0.69 million deaths; over two-thirds of them were in sub-Saharan Africa [1]. In 2018, an estimated 228 million cases and 0.41 million deaths due to malaria occurred worldwide, more than 90% of which occurred in sub-Saharan Africa [2]. Because of the broad overlap of endemic regions and the great numbers of people infected, especially in sub-Saharan Africa and Southeast Asia, there is a high risk that people are coinfected with HIV and malaria parasites. Infection with either HIV or *Plasmodium* pathogens has a profound impact on the immune system of human hosts and induces distinct immune responses. The alteration in immune function induced by infection with one pathogen may greatly modulate immune protection against the other. Therefore, to develop a more effective intervention strategy to control the spread of these diseases, a comprehensive understanding of the interaction between HIV infection and malaria is urgently needed.

It has been reported that 11.7% and 25.9% of HIV-positive patients in Ghana [3] and Mozambique [4] and 27.8% of HIV-positive pregnant women [5] are infected with *Plasmodium* parasites, respectively, and these data also showed that concomitant HIV infection resulted in an increased risk and severity of malaria infection. However, the impact of malarial infections on the pathogenesis of HIV infection is not yet fully understood. Epidemiological studies concerning this question did not reach a consistent conclusion. Some have suggested that malaria may promote HIV replication and accelerate the decline in CD4^+^ T lymphocytes [6–8]. However, one cohort study suggested that children who were infected with HIV through vertical transmission and contracted malaria after they were born had longer survival times than HIV-positive children who were free from malaria [9]. Although many factors, including host genetics, age, parasite exposure, parasite strain and duration of the infection, may influence the progression of HIV-malaria coinfection, these inconsistent observations are likely due to differences in the order of these two infections, which was not investigated in previous coinfection animal model studies. In most malaria-endemic areas (in “real world”), HIV would be acquired when individuals become sexually active. At this time, most young adults would have already developed substantial malaria immunity, or have a chronic malarial infection before obtaining HIV. But it is still possible for some individuals to get HIV before infecting malarial parasites, for example, HIV-infected children who obtained the virus through vertical transmission and are infected with malaria later, or HIV-infected persons who travel to malaria-endemic areas.

Thus, we hypothesized that contraction of *Plasmodium* parasites before or after HIV infection might influence the outcome of HIV disease. In this work, we studied the pathological and immunological effects of pre-existing or subsequent malaria on simian immunodeficiency virus (SIV) infection under controlled conditions in an established Chinese rhesus macaque coinfection model with SIVmac251 and *Plasmodium cynomolgi* (*P. cynomolgi* or Pc) [10, 11] and focused on the impact of malaria on the ultimate result of SIV infection. Our results demonstrate that the infection order of the two pathogens did have different effects on the outcome of SIV disease. We found that malaria contracted after SIV infection resulted in sustainably higher proportions of CD4^+^CD28^high^CD95^high^ central memory T cells, induced SIV-specific cellular immune responses, restored the diversity of the SIV-specific T cell receptor (TCR) repertoire and substantially improved the survival of the coinfected animals. In contrast, malaria infection initiated prior to SIV infection induced an increased number of SIV-targeted CD4^+^CCR5^+^ T cells and resulted in higher plasma viral loads, as previously reported.

## Results

### Establishment of an animal model for HIV and *Plasmodium* coinfection

**Fig. 1:**
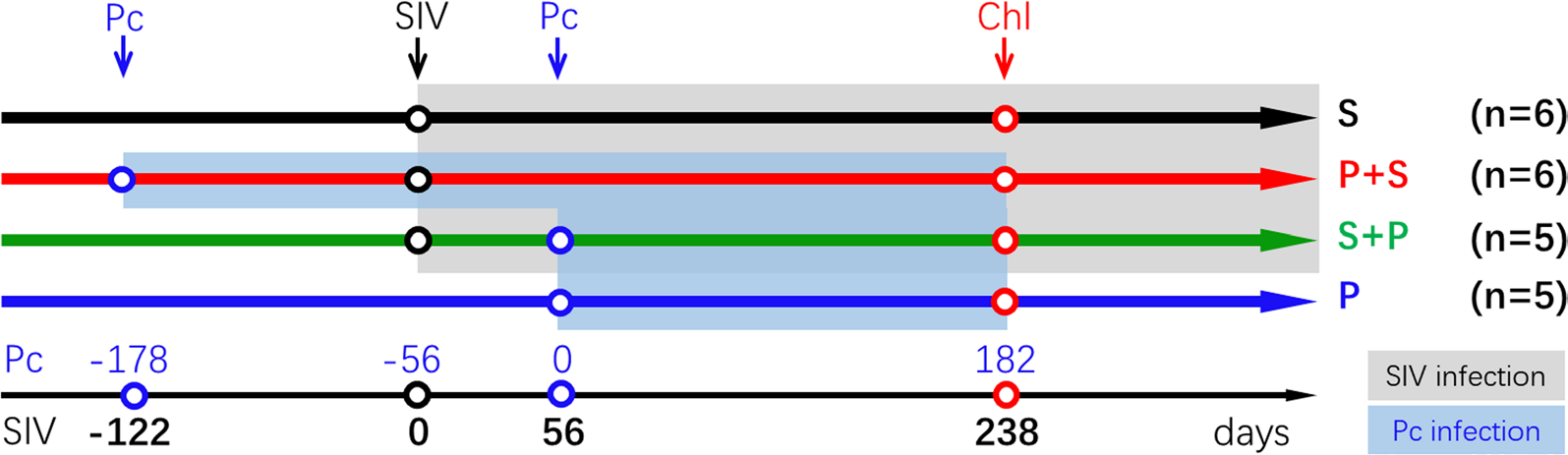
Schematic of the experimental infection of Chinese rhesus macaques with blood-stage *Plasmodium cynomolgi* and Simian immunodeficiency virus (SIVmac251). *P. cynomolgi* (Pc) and SIVmac251 (SIV) infections are indicated by blue and black circles, respectively. Pc-infected animals were treated with chloroquine phosphate (Chl, red circles) for three days at the indicated times. The gray area indicates the SIV phase, and the light blue area indicates the Pc malaria phase

The patterns of coinfection are complicated in sub-Saharan Africa, Southeast Asia, and South America, which have a high incidence of new HIV infections and are endemic for *P. falciparum* or *P. vivax* malaria. Most individuals coinfected with HIV and malarial parasites in these regions were exposed to *Plasmodium* before infection with HIV as adults. Some contracted malaria after HIV infection, such as infants through vertical infection or HIV-positive patients who migrated from nonmalaria regions into malaria-endemic regions. There are cases in which individuals acquire HIV and *Plasmodium* almost simultaneously or contract malaria several times. We simplified the patterns into two scenarios: malaria contracted before or after HIV infection. To more closely model natural human exposure, animals were first inoculated with *P. cynomolgi* and then with SIV at the chronic phase of malaria (P + S), or first with SIV and then *P. cynomolgi* after the set point [12] viral load was established (S + P). Animals infected with SIV alone (S) or *P. cynomolgi* alone (P) served as controls (Fig. 1). After inoculating SIV or *Plasmodium* as scheduled, we examined the plasma SIV viral loads during the course of SIV infection and parasitemia during malaria. Animals in both the P and S + P groups displayed the expected malaria disease course with typical symptoms such as fever, anemia and parasitemia that have been reported in our previous publication [13]. SIV load in peripheral blood was detected in animals in the S, P + S and S + P groups and reached the peak level at two weeks after SIV inoculation (Fig. 3A). These data demonstrate that singly infected and coinfected models were successfully established.

**Fig. 2:**
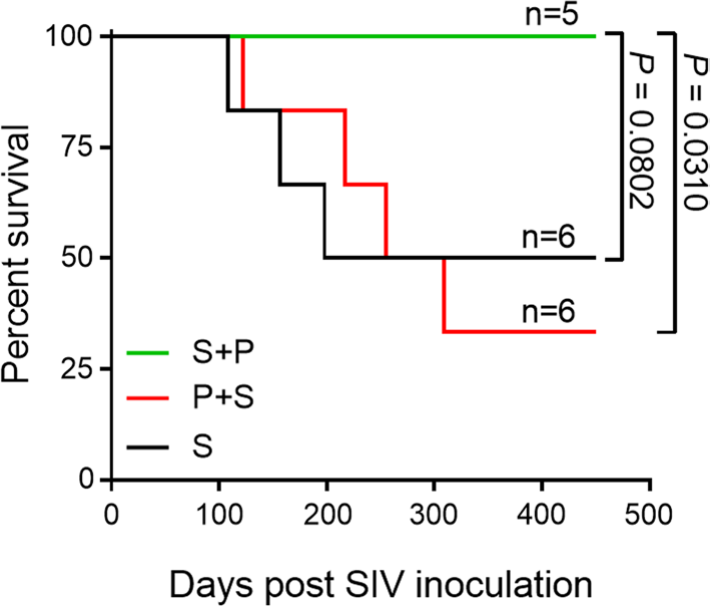
Survival rates of monkeys from the S, S + P and P + S groups. The median survival times of the S and P + S groups were 324 days and 282 days, respectively. Statistical significance of the difference in survival between groups was determined by the log-rank (Mantel-Cox) test

**Fig. 3:**
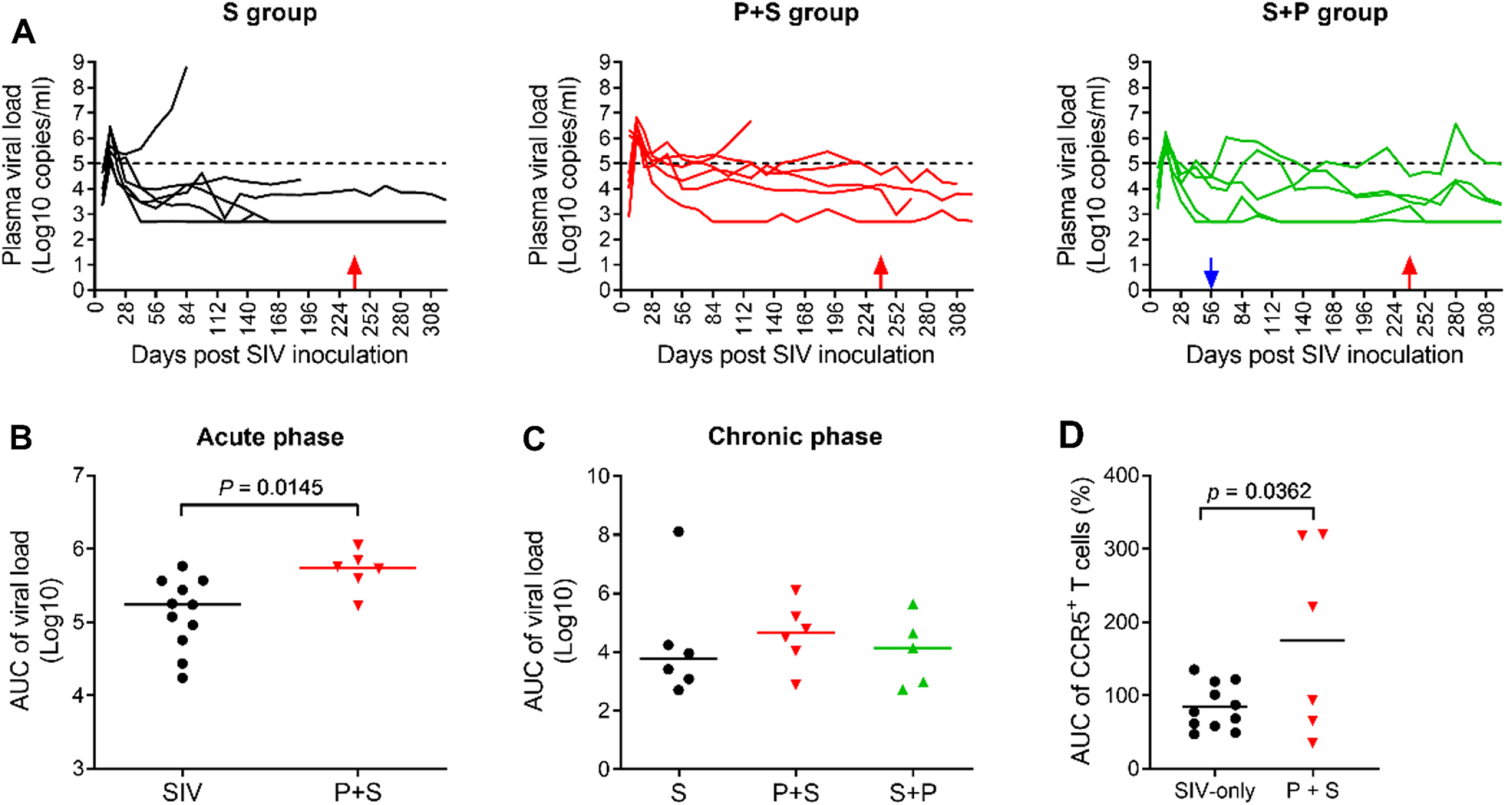
Plasma viral loads in SIV-infected animals. **A** Plasma viral loads of individual monkeys in the three SIV-infected groups. Blue and red arrows indicate the times of Pc inoculation and chloroquine treatment, respectively. **B** Area under the curve unit (AUC/day) of plasma viral loads in SIV infected monkeys: S group plus S + P group versus P + S group (before Plasmodium infection) during the acute phase (days 0–56). **C** AUC/day of plasma viral loads during the chronic phase of SIV infection (all surviving animals from days 70–322). The differences between groups were not significant. **D** AUC of percentage of peripheral CCR5^+^CD4^+^ T cells from SIV-infected macaques: S group plus S + P group (before Plasmodium infection) versus P + S group during the days 0–7. Note: During the acute phase of SIV infection, the monkeys either in S group or in S + P group had no Plasmodium infection, therefore they were pooled together as SIV-only group (Fig. 3B, D); but during chronic phase of SIV infection, the monkeys in S group had no malaria, and the monkeys in S + P group had malaria, therefore they could not be pooled together (Fig. 3C). Acute phase: Day 0–56; Chronic phase: After Day 56

### Malaria before and after SIV infection modulates animal survival in different ways

To test whether the order in which malaria and SIV occur has any effect on animal survival, we designed a model in which animals were infected with *Plasmodium* before SIV (P + S) or SIV before *Plasmodium* (S + P), while single infections with *Plasmodium* (P) or SIV (S) served as controls. The infected animals were monitored regularly until the experiment was terminated at the end point. Four hundred and fifty days after SIV infection, we observed that malaria contracted before or after SIV infection had different effects on the ability of animals to survive SIV infection. While all five animals with subsequent malaria infection (S + P) survived SIV infection (*P* = 0.08 compared with the S group), four out of 6 animals in the P + S group died between days 122 and 309 after SIV infection (median survival of 282 days, *P* = 0.03 compared with the S + P group) (Fig. 2). Furthermore, three out of 6 animals in the S control group died between days 108 and 198 following SIV infection (median survival of 324 days, *P* = 0.88 compared with the P + S group). These results suggest that subsequent malaria infection could enhance the survival of SIV-infected animals and call for further investigation.

### Pre-existing malaria infection prior to SIV infection promotes viral replication

We examined the plasma SIV viral load, a marker of AIDS progression, in the course of SIV infection. Generally, the coinfected animals had elevated viral loads compared with SIV-singly infected animals, but at different levels in the pre-existing and subsequent malaria groups.

During the acute phase of SIV infection (days 0 to 56), the median plasma viral loads presented as the area under the curve per day (AUC/day) in animals of the P + S group were 0.50 logs higher than those in the SIV-infected animals (including 11 macaques of the S and S + P groups in which animals were not infected with Pc at the time) (Fig. 3B).

During the chronic phase of SIV infection (from days 70 to 322), pre-existing malaria (P + S group) caused median viral loads to elevate by 0.97 logs of AUC/day compared with the S group (ns), while subsequent malaria elevated the viral load by 0.73 logs of AUC/day in the S + P group (ns). Notably, there was no significant difference in viral load between the P + S group and the S + P group (Fig. 3C), suggesting that the disparity in SIV disease progression and animal survival observed in the coinfection model is independent of the plasma viral load.

### Pre-existing malaria infection prior to SIV infection induces more CD4^+^CCR5^+^ T cells

Examination of peripheral blood T lymphocyte levels revealed that the mean CD4^+^ T cell counts declined at similar rates in the three groups of SIV-infected animals when most of them were still alive (before day 168 of SIV infection). An obvious increase in the mean CD4^+^ T cell count was observed in the P group after the acute phase of Pc infection (Additional file 1: Fig. S1A). The mean CD8^+^ T cell numbers in the S and P + S groups remained steady. There was a slight decrease in CD8^+^ T cells during the acute phase of Pc infection in the P and S + P groups, and CD8^+^ T cells recovered thereafter (Additional file 1: Fig. S1B). In groups S and P + S, animals with lower levels of mean CD4^+^ or CD8^+^ T cells died during the early stage, while animals with higher levels of T cells survived. The gradual reduction in CD4^+^ T cells and steady CD8^+^ T cells resulted in decreased CD4^+^/CD8^+^ T cell ratios in the SIV-infected animals (S, S + P and P + S), but there were significant increases in the CD4^+^/CD8^+^ T cell ratio during the acute phase of malaria in both the P and S + P groups (Additional file 1: Fig. S1C).

We further examined the frequency of CD4^+^CCR5^+^ T cells, which are considered the major target cells of SIVmac251 [14], in the peripheral blood of the four groups of animals. A significant increase in this T cell subpopulation was observed in the P + S group during the early phase of SIV infection in comparison to animals infected with SIV alone (Fig. 3D, Additional file 1: Fig. S1D). After Pc infection, there was no significant difference in the level of CD4^+^CCR5^+^ T cells between the S group and the S + P group (Additional file 1: Fig. S1D). These data indicate that pre-existing Pc infection induced more CD4^+^CCR5^+^ T cells and may explain why viral replication is enhanced in this group.

### Subsequent malaria infection induces a sustainably higher proportion of central memory CD4^+^ T cells

**Fig. 4:**
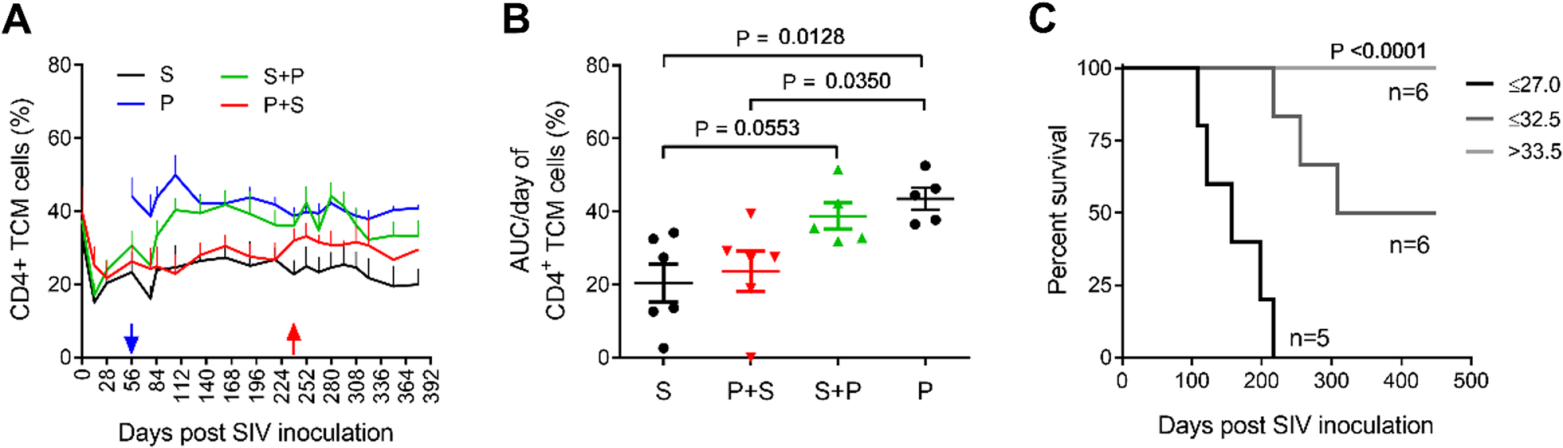
Correlation between the frequency of TCM and survival. **A** Frequency of CD4^+^CD28^high^CD95^high^ TCM during the course of SIV, Pc infection or coinfection. The data were from all the animals that survived at the assay time point. **B** AUC/day of TCM frequency during the chronic phase of Pc infection (days 84–238). One-way ANOVA multiple comparisons, *P* = 0.0059. **C** The relationship between TCM frequency and prognosis. Kaplan–Meier curves for peripheral blood TCM frequency (AUC/day) during the chronic phase of Pc infection (days 84–238) and survival of SIV-infected animals. The animals were divided into three groups according to percentiles of AUC/day of TCM frequency in CD4^+^ T cells. Log-rank (Mantel-Cox) test, *P* < 0.0001. The data represent all seventeen SIV-infected monkeys in the S, S + P, and P + S groups

Both SIV and HIV infections are often accompanied by massive depletion of effector memory CD4^+^ T cells (TEMs), which can be repopulated by central memory (TCM) precursors. We analyzed the memory subsets of CD4^+^ T cells according to the expression of the CD4, CD28 and CD95 molecules on the cell surface (Additional file 1: Fig. S2). During the acute phase of SIV infection, the percentage of CD28^+^CD95^+^ TCMs in SIV-infected macaques decreased sharply in the three SIV-infected groups, then increased slightly but was maintained at the lower level and did not reach the level before SIV infection in the S and P + S groups (Fig. 4A). In contrast, the proportion of TCMs in animals in the S + P group increased significantly during the chronic phase of subsequent malaria and maintained that level until the end of the experiment (Fig. 4A, B).

A clear survival advantage was observed for animals with higher TCM levels based on the quartile rank of the TCM percentage (*P* < 0.0001, Fig. 4C). Seven out of eight animals with a lower TCM percentage (on day 133) eventually died from SIV infection. These results indicate that malaria infection after SIV infection could increase the proportion of TCM, which might have contributed to the survival of animals in the S + P group.

### Subsequent malaria enhances the SIV-specific cellular immune response

**Fig. 5:**
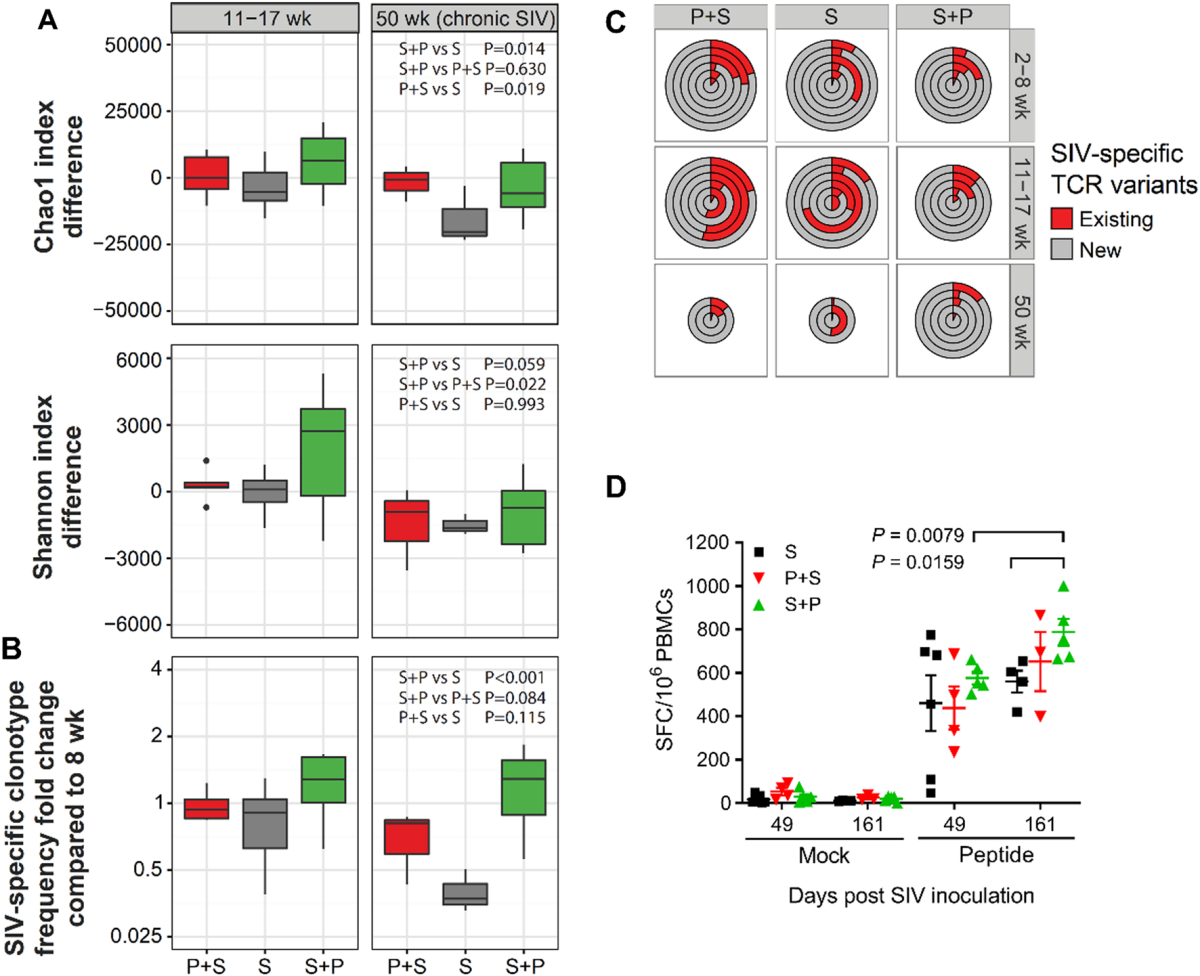
T-cell repertoire diversity and SIV-specific immune response in malaria- and SIV-infected monkeys. **A** Diversity index differences with respect to the 8th week of SIV infection (corresponding to malaria introduction in the S + P group). Repertoire diversity was restored in the S + P group but not in the P + S group. A significant difference between the S + P and P + S groups was observed for the Shannon diversity index. *P*-values computed using ANOVA. **B** Ratio of SIV-specific clonotype frequency with respect to the control point (8th week of SIV infection, corresponding to malaria introduction in the S + P group). The S + P group, but not the P + S group, was characterized by a persistently higher SIV-specific clonotype frequency. *P*-values were calculated using ANOVA. **C** New SIV-specific clonotype production. Fractions of existing (detected at previous sampling point) and new SIV-specific clonotypes shown for each monkey in the P + S, S and S + P groups. A significant increase in new SIV-specific clonotype production was observed in the S + P group during the whole period of *Plasmodium* infection (at 11–17 weeks and 50 weeks post SIV infection). **D** Frequency of IFN-g-producing SIV Gag-specific PBMCs. Number of IFN-γ-producing PBMCs against pooled SIV Gag peptides analyzed by ELISPOT. The sample sizes were 6, 4 and 5 on day 49 and 4, 3, and 5 on day 161 of the S, P + S and S + P groups, respectively. SFC: spot-forming cells

To evaluate the cell-mediated immune response, we determined the frequency of IFN-γ-producing cells in peripheral blood mononuclear cells (PBMCs) using the ELISPOT assay against pooled SIV Gag peptides. The animals in the S + P group produced significantly more SIV-specific cells producing IFN-γ after Pc inoculation (S + P, day 161 vs day 49 (*P* = 0.0079); or day 161, S + P vs S (*P* = 0.0159)); the P + S group had no significantly enhanced SIV-specific cell reaction compared with the S group at either measured time point (Fig. 5D). No significant differences were observed in the levels of total SIV-specific antibodies among the three SIV-infected groups (Additional file 1: Fig. S3). These data indicate that subsequent malaria evoked mainly cellular rather than humoral immune responses against SIV.

### Subsequent malaria restores the diversity of the SIV-specific TCR repertoire

T cells have been demonstrated to play a critical role in controlling disease progression in both the acute and late stages of SIV infection. Previous studies demonstrated that malarial infection profoundly altered the T cell receptor (TCR) β repertoire [15]; therefore, we sought to know the effects in our coinfection model. We analyzed SIV-specific cellular immunity at the molecular level by next-generation sequencing of the TCR. We calculated two indices to estimate the diversity of the TCR repertoire: the Chao1 index, which estimates the total TCR repertoire based on the discovered rare clonotypes (singletons), and the Shannon index, which quantifies the entropy of clonotype frequency distributions. After SIV infection, the Chao1 index (Additional file 1: Fig. S4A left) and Shannon index (Additional file 1: Fig. S4A, right) gradually decreased. Compared with at week zero, at the 50th week, the significant reduction in the indices indicated that SIV infection caused the TCR repertoire to shrink in the surviving macaques (Additional file 1: Fig. S4A). On the other hand, *Plasmodium* infection increased the Shannon index at the acute malaria phase but not the total repertoire diversity (Additional file 1: Fig. S4B). The increase in Shannon index was a result of attenuation of dominant clonal expansions or malaria-mediated broad clonal expansion. The decrease in the relative frequency of expanded clonotypes occurred upon acute malaria infection (week 3 post-malaria introduction) (Additional file 1: Fig. S4C). The change in diversity indices at the 8th week of SIV infection (corresponding to malaria introduction in the S + P group) was compared between the coinfection groups (Fig. 5A). The Shannon diversity index was observed to be significantly different between the S + P and P + S groups. Repertoire diversity was restored in animals that received *Plasmodium* injection after SIV infection (S + P) but not malaria infection prior to SIV infection (P + S).

We identified SIV-specific clonotypes in our data based on the tetramer sorting data by Price et al. [16] and characterized the properties of SIV-specific clonotypes. The amino acid sequences of SIV-specific clonotypes tended to become less hydrophobic (decreased GRAVY index), and the DNA sequences of the CDR3 NDN region lengthened compared with the control repertoire (Additional file 1: Fig. S5B). At the acute phase of malaria (three weeks after parasite inoculation in the P group), the TCR repertoires were characterized by decreased hydrophobicity (GRAVY index) and increased NDN size compared with the repertoires before Pc inoculation (Additional file 1: Fig. S5A). These changes in hydrophobicity and NDN size suggest that Pc infection evoked antigen-specific T cell expansion extensively and increased polyreactive clonotype frequency.

We also compared the fold change in their frequency with the control point (corresponding to malaria introduction in the S + P group 8 weeks after SIV infection). Malaria introduction after (S + P), but not before SIV infection (P + S), was characterized by a persistently higher SIV-specific clonotype frequency, unlike in the S group, which remarkably demonstrated a sharp decrease (Fig. 5B). Fifty weeks after SIV infection, SIV-specific clonotypes decreased in the P + S and S groups (P + S vs S + P, *P* = 0.084; S vs S + P, *P* < 0.001). These results suggest that Pc infection elicited not only parasite-specific [13] but also SIV-specific T cell responses.

When comparing fractions of existing (detected at previous sampling points) and new SIV-specific clonotypes, there were more new SIV-specific clonotypes continuously generated in the S + P group than in the S or P + S group (Fig. 5C). Significant increases in the SIV-specific clonotype were observed in the S + P group at 11–17 weeks (corresponding to the acute phase of malaria in this group) and 50 weeks (corresponding to the chronic phase of malaria in this group) post-SIV infection, suggesting that the subsequent malaria primes T cells in accordance with SIV mutation. These results demonstrate that subsequent malaria not only promoted the generation of SIV-specific cytotoxic T cells but also boosted and maintained a relatively high production of SIV-specific clonotypes, which probably contributed to the survival of the animals in the S + P group.

## Discussion

Since AIDS and malaria coexist in many parts of the endemic areas, coinfection in humans with the two pathogens is highly likely to occur. There is an urgent need to illustrate the physiological and immunological aspects of the interaction between the two infections. In our current controlled study, we demonstrated that blood-stage *P. cynomolgi* infection initiated before and after SIV infection had different impacts on the course and prognosis of SIV infection. Pre-existing parasites accelerated SIV progression as previously reported, but subsequent malaria infection improved the survival of SIV-infected animals by expanding CD4^+^ TCM and enhancing virus-specific T cell immunity. This survival benefit was not attributed to the less severe malarial disease in S + P group. Instead, malaria in S + P group was more severe than in P group [13] and P + S group (data not shown). This is consistent with a previous report that HIV-infected children who obtained the virus through vertical transmission and were infected with malaria later had longer survival times than HIV-positive children without malaria [9].

Animals coinfected with malaria and SIV had an elevated plasma viral load compared with SIV-singly infected animals, but the animals in the P + S group had a higher viral load than those in the S + P group in the present study. It has been shown that *Plasmodium* antigens can activate CD4^+^ T cells and enhance HIV replication in vitro [17–19]. Previous epidemiological reports demonstrated that HIV-malaria-coinfected humans had an elevated viral load [6, 7, 20, 21]. We also found that more CD4^+^CCR5^+^ T cells were induced in the P + S group than in the S group, which is consistent with a report by Koehler et al. [22]. The elevated proportion of CD4^+^CCR5^+^ T cells induced by *Plasmodium* parasites might provide more target cells for SIV replication and account for the higher viral load in the pre-existing malaria group than in the subsequent malaria group.

HIV/SIV infection is characterized by the persistent loss of CD4^+^ T cells, leading to immunodeficiency and subsequently AIDS/SAIDS. One of the reasons for the loss of CD4^+^ T cells may be associated with a defective restoration of either the quantity [23, 24] or the quality [25] of the CD4^+^ TCMs. As CD4^+^ TCMs play a pivotal role in T cell homeostasis and in maintaining the TEM compartment, they are required for clinical immune competence [23, 26]. The regenerative capacity of the central-memory compartment of CD4^+^ T cells is important for maintaining a disease-free state and long-term survival in both SIV-infected macaques [27–29] and HIV-infected humans [24, 30]. Previous studies have demonstrated that loss of TCM cells might predict rapid progression in SIV-infected pigtail macaques [27], as indicated here that the loss of TCMs was correlated with the survival of animals in the S group (Fig. 4C). On the other hand, *Plasmodium* infection induces an expansion of activated naïve/memory T cells and differentiation into central memory T cells in *P. vivax*-infected humans [31] and *P. chabaudi*-infected mice [32]. Our results show that *P. cynomolgi* malaria also induced the generation of TCM cells in animals in the P and S + P groups (Fig. 4A, B). These factors might benefit the survival of the animals, as demonstrated by a clear survival disadvantage in animals with lower TCM levels in the S and P + S groups (Fig. 4C).

In the present study, a stronger SIV-specific cellular immune response was induced in animals in the S + P group than in the S or P + S groups, as shown in the ELISpot assay (Fig. 5D), suggesting that these SIV-specific T cells may contribute to the sustainably higher proportion of TCMs in the S + P group. TCR sequencing data also indicated that more SIV-specific T cell clones were generated during the acute malaria and conserved to the late chronic phase of SIV infection (Fig. 5B), and the production of the SIV-specific clonotype was faster (Fig. 5C) in the S + P group than in the S or P + S groups, suggesting that the subsequent malaria may have induced the generation of T cells in response to SIV mutation. Furthermore, richer T cell profiles enhance the immune response to HIV/SIV infection and benefit hosts against opportunistic infections that are common in patients or animals with AIDS.

The pool of resting CD4^+^ T cells, including CD4^+^ central memory T (TCM) and effector memory T (TEM) cells, is a major viral reservoir because antiretroviral therapy (ART) does not affect the provirus within these cells [33]. Activation of resting T cells and higher virus burden caused by malaria might have the potential to enlarge the latent SIV reservoir. However, our previous study has shown that subsequent malaria reduced the volume of the viral reservoir in SIV-infected macaques during ART [34]. In the current study, activation of resting T cells and enhanced SIV-specific immune response caused by malaria after SIV infection might be favorable to eradicating the latent SIV infection and reducing the reservoir which, in turn, benefits the survival of animals in the S + P group.

It is worth noting that Ryan-Payseur et al. did a similar study [35], using Chinese rhesus macaques co-infected with SHIV (simian-human immunodeficiency virus) and *P. fragile* (a simian malarial parasite which induces symptoms that are similar to those of *P. falciparum* infection in humans) to compare the effects of simultaneous acute co-infection with those of chronic SHIV infection with subsequent malaria (similar to the situation of our S + P group) on the disease progression. The authors concluded that simultaneous acute co-infection resulted in increasing CD4 + T-cell depletion, profound lymphoid depletion or destruction, and even necrosis in lymph nodes and spleens, therefore promoting parasite replication and inducing fatal virus-associated malaria (but the situation of simultaneous acute co-infection with both pathogens should be rare in the “real world”), and that chronic SHIV-infection with subsequent malaria induced different defense mechanisms involving a unique 200-fold expansion of interleukin 17^+^/interleukin 22^+^ T effector cells with profound Th1 suppression that were coincided with development of immunity against fatal virus-associated malaria without accelerating SHIV disease. But they did not observed a survival advantage in the monkeys with chronic SHIV infection and subsequent malaria, potentially due to their relatively short period of observation. Until now, our current study belongs to the first time in comparing the effects of pre-existing malaria and subsequent malaria on the disease progression of SIV infection through observing a sufficiently long period, central memory CD4^+^ T cell and SIV-specific T cell immune responses, including SIV-specific TCR repertoire diversity. Therefore we are able to observe the survival advantage of the S + P group.

It should be noticed that we have excluded the macaques that express the MHC class I alleles Mamu-A*01, Mamu-B*08 or Mamu-B*17, the common genotypes associated with the ability to control viral replication. Therefore, our macaque screening strategy has basically excluded the elite controllers in the experiments that may affect the results (see the Materials and Methods section).

Taken together, these results suggest that malaria contracted after SIV infection stimulates naïve T cells to proliferate and differentiate into TCMs, enriches the functionally diverse repertoire of T cells, enhances virus-specific T cell immunity, and finally, improves the survival of infected hosts. On the other hand, malaria contracted before SIV infection did not exert the effects that were seen in the S + P group on animals in the P + S group but provided more target cells for SIV infection by activating T cells and generating more CCR5^+^ effector T cells. Thus, animals in the P + S and S groups showed poor prognosis.

There are some limitations in our current study: (1) the malarial parasite (Pc) we used is a relatively benign form in macaques, which might not represent the *P. falciparum* in humans that is dominant in sub-Saharan Africa; (2) the tested samples only covered the peripheral blood, without gut tissues and lymph nodes; (3) our animal sample is relatively small due to economic and ethical aspects of using nonhuman primates.

## Conclusions

In conclusion, our current study suggests that malaria and SIV dual infection modulate the severity and prognosis of SIV infection in different ways depending on the sequence of the two infections. Pre-existing malaria infection promotes SIV replication, which may lead to faster progress in SIV disease in coinfected animals, while subsequent malaria enhances the CD4^+^ TCM response and virus-specific T cell immunity, resulting in the improved survival of SIV-infected hosts. The underlying mechanism needs further study. These observations provide evidence that may reconcile the inconsistent conclusions drawn from previous human epidemiological studies and will guide future studies on HIV and malaria coinfection in humans.

## Materials and methods

### Ethics statement

Animals were housed in the facilities of the Non-Human Primate Animal Center of the Guangzhou Institutes of Biomedicine and Health (GIBH). All experimental procedures were conducted in the animal facility and in strict compliance with the GIBH Protocol for Institutional Animal Care and Use Committee (IACUC), which is in accordance with the recommendations of the Weatherall report, “The use of non-human primates in research”. Each animal was housed in a separated cage, received standard primate feed and fresh fruit or eggs daily, and had free access to water. The animals were monitored daily for body temperature, weekly for body weight and routine blood biochemistry and monthly for physical fitness under the supervision of the veterinarians in charge of the animal facilities. All efforts were made to minimize suffering, including efforts to improve housing conditions (e.g., minimizing the volume of blood draw, 12:12 light: dark schedule). Euthanasia was performed when the symptoms of disease were developed (e.g., for macaques when the biological markers indicated progression towards the disease, such as significant CD4^+^ T cell decline and increases in viremia). Euthanasia was performed by i.v. injection of a lethal dose of pentobarbital. Animal experimental protocols were approved by the Laboratory Animal Committee (LAC) of GIBH and the GIBH IACUC (No. 2007013).

### Animals

Twenty-two adult Chinese rhesus macaques (*Macaca mulatta*), 5–6 years of age, were used in this study. They were born and raised in captivity in our collaborative nonhuman primate animal center. Among them, eleven were male and eleven were female, and they were divided as equally as possible into each group. All animals were free of B virus, D-type simian retrovirus, simian T-lymphotropic virus type 1 and SIV. The macaques selected for this study did not express the MHC class I alleles Mamu-A*01, Mamu-B*08 or Mamu-B*17, the genotypes associated with the increased ability to control viral replication [36–40].

### *P. cynomolgi* and SIV infection procedures

To design an animal model that more closely mimicked natural human exposure, macaques were divided into four groups with different inoculation procedures: P + S (n = 6), S + P (n = 5), S (n = 6) or P group (n = 5) (Fig. 1). SIV was given by intravenous (i.v.) inoculation of 300 TCID_50_ SIVmac251 virus. Blood-stage *P. cynomolgi Berok strain* (*P. cynomolgi,* Pc) malaria was initiated by i.v. injection of 1 × 10^7^ *P. cynomolgi*-infected red blood cells (iRBCs) from a donor macaque. For the P + S group, the animals were infected with SIV on day 122 after Pc inoculation when the malaria progressed into the chronic phase characterized by low levels of parasitemia and a lack of fever. For the S + P group, the animals were infected with Pc on day 56 after SIV infection when the set point viral load was established. Two groups of animals were infected with either SIV alone (S group) or Pc alone (P group) as controls.

The Pc-infected animals were monitored for parasitemia by thin blood smears. When the parasitemia reached 10% (infected erythrocytes among total red blood cells), the animals were treated with 5 mg artesunate i.v. to prevent death by severe malaria. The three groups of Pc-infected animals were treated orally with chloroquine phosphate for three days (total dose = 67 mg/kg) starting on day 238 post-SIV infection to cure malaria. The animals in the S group were also treated with chloroquine phosphate following the same regimen as a control for the effects of the drug treatment. SIV-infected animals were kept under close veterinary observation and were humanely euthanized upon a final diagnosis of AIDS. The infected animals were monitored daily for 450 days after SIV infection when the experiment was terminated.

### Viral detection assays

Plasma viral RNA levels were determined using a SYBR Green real-time PCR protocol as described in our previous report [41]. The viral RNA was purified with a QIAamp Viral RNA Mini Kit (Qiagen, Valencia, CA), and a real-time PCR assay was carried out with a one-step SYBR Green Real-Time PCR Kit (Qiagen). The nominal threshold for this assay was 100 viral RNA copy equivalents/ml plasma.

### Flow cytometry

T cell subpopulations in peripheral blood were analyzed by flow cytometry. The following conjugated antibodies were purchased from BD Biosciences (San Diego, CA, USA): anti-CD4-FITC, anti-CD4-PE-Cy7 and anti-CD4-PerCP (L200); anti-CD8-APC-Cy7 (RPA-T8); anti-CD45-PE (DO58-1283); anti-CD3-PerCP and anti-CD3-Pacific Blue (SP34-2); anti-CD28-FITC (CD28.2); anti-CD95-APC and anti-CD95-PE cy5 (DX2); and anti-CCR5-PE (3A9). The conjugated antibodies anti-CD8-APC and anti-CD8-PE (B9.1) were obtained from Immunotech SAS (Marseille Cedex, France). The number of lymphocytes in peripheral blood samples was determined using the BD TruCOUNT kit (BD Biosciences, San Jose, CA, USA). Cell surface marker staining for flow cytometry was performed as follows: 50 μl of EDTA-treated whole blood was incubated with antibodies at room temperature (RT) for 20 min in the dark, and red blood cells were lysed in Lysing Buffer (BD Pharmingen) at RT for 10 min. Following a wash with PBS containing 2% FBS, the cells were analyzed by flow cytometry on either a FACSCalibur (BD Biosciences) or FACSAria (BD Biosciences) system. The data were analyzed using FlowJo software (Tree Star, Inc., USA).

### IFN-γ ELISPOT assay

The ELISPOT assay was performed following the previously described protocol with minor modifications [42]. Briefly, 96-well Immobilon-P membrane plates (Millipore Corporation, Billerica, MA, USA) were coated with an anti-monkey IFN-γ monoclonal antibody (BD Biosciences) overnight at 4 °C. The plates were then washed with PBS and blocked with R10 medium (RPMI 1640, 0.05 mM 2-mercaptoethanol, 1 mM sodium pyruvate, 2 mM L-glutamate, 10 mM HEPES, and 10% fetal bovine serum) for 2 h (h) at 37 °C. The medium was discarded, and freshly isolated PBMCs were added at a concentration of 4 × 10^5^ cells/well. Pools of overlapping SIVmac239 peptides (provided by the NIH AIDS Research and Reference Reagent Program) representing the entire amino acid sequence of Gag were added at a concentration of 2 μg/ml per peptide. Cells were then incubated for 20–24 h at 37 °C in 5% CO_2_. The plates were washed with PBST (PBS containing 0.05% Tween-20), and a biotinylated anti-IFN-γ polyclonal detector antibody (BD Biosciences) was added. The plates were incubated overnight at 4 °C. After washing, streptavidin–alkaline phosphatase was added to the plates and allowed to incubate for 2 h at 37 °C. After washing, the color was developed by incubating in NBT/BCIP (Pierce, Rockford, IL) for 10 min. Spots were counted with an ELISPOT reader (Bioreader 4000, BIOSYS, Germany), and data were reported as numbers of spot-forming cells (SFCs) per million PBMCs.

### PBMC isolation and DNA extraction

PBMCs were isolated using LymphoPrep (Axis-shield, Dundee, Scotland, UK) with slight modification to the manufacturer’s instructions. Briefly, 10 ml of blood was diluted with an equal volume of PBS, loaded carefully onto 5 ml of LymphoPrep (sample to LymphoPrep media, 2:1) and centrifuged at 600×*g* for 20 min. The thin PBMC layer at the sample/medium interface was carefully aspirated and transferred to a new tube and washed twice with PBS at 300×*g* for 10 min.

Rhesus macaque genomic DNA was prepared from 5 × 10^6^ cells from each sample, which was sufficient for analyzing the diversity of the TCR β-chain. The DNA was extracted using the PureLink Genomic DNA Mini Kit (Life Technologies, Cat. No: K1820-00) according to the manufacturer’s instructions.

### Multiplex PCR amplification of the TCR-β CDR3 region

To generate a template library for the Illumina MiSeq sequencer, multiplex PCR was designed to amplify rearranged TCRβ CDR3 regions from genomic DNA based on our previously established method with newly designed primers [43]. The assay utilized a suite of 37 forward (VF) primers specific for functional TCR Vβ segments and 13 reverse (JR) primers specific for Jβ segments (Table 1). Each VF or JR primer contains a universal forward or reverse primer sequence and five random nucleotides at their 5′-ends, respectively. The PCRs (50 μl) were configured with 25 μl of 2 × QIAGEN Multiplex PCR Master Mix, 5 μl of Q-Solution (QIAGEN), 1.5 μl of VJ primer pool (75 nM for each unique TCR primer) and up to 1 μg of gDNA. The amplification protocol was as follows: 15 min at 95 °C, 20 cycles of 30 s at 94 °C, 90 s at 63 °C, and 30 s at 72 °C, followed by a final extension cycle of 15 min at 72 °C on a PCR Express Thermal Cycler (Thermo Hybaid, California). To sample as many rearranged TCR CDR3 loci as possible, every sample was amplified by PCR in 12–20 replicate wells. The PCR products of the same sample were pooled and purified by size selection using magnetic beads. The libraries were prepared using Illumina® TruSeq Nano DNA Sample Prep Kits (HT) (Illumina, California) according to the manual with some modifications. Briefly, the purified amplicons were end-repaired, adenylated at the 3’ ends and ligated with adapters (each sample was barcoded). Then, the products were PCR amplified for 8 cycles. The final library was sequenced on a MiSeq with a MiSeq Reagent Kit v3, 600 Cycles (Illumina, California), generally using a read length of 300 bp.

**Table 1:** The primers used for amplifying the TCRβ segments

Code	Sequence
BVF01	GCAGTCGTGCAGCAAGTTTTNNNNNCAGGAAGTGATCTTGCGGTGTG
BVF02	GCAGTCGTGCAGCAAGTTTTNNNNNMCCTTAAATGTGAACAAAATCTGGGTC
BVF03	GCAGTCGTGCAGCAAGTTTTNNNNNAATGTGAACAAAATCTGGGCCAT
BVF04	GCAGTCGTGCAGCAAGTTTTNNNNNTACACCAAAGCACCTGGTCATGGGA
BVF05	GCAGTCGTGCAGCAAGTTTTNNNNNTGAGTTACGCAGACACCAAGACACC
BVF06	GCAGTCGTGCAGCAAGTTTTNNNNNGATGCTCTCCTATCTCTGGGCAC
BVF07	GCAGTCGTGCAGCAAGTTTTNNNNNCAGTGTGCCCAGGATATGAACCA
BVF08	GCAGTCGTGCAGCAAGTTTTNNNNNAAGTGTGCCCAGGATATGAACCA
BVF09	GCAGTCGTGCAGCAAGTTTTNNNNNCAGTGTGCCCAGGATATGAGCCA
BVF10	GCAGTCGTGCAGCAAGTTTTNNNNNCCGCCCTTTATTGGTACCGACA
BVF11	GCAGTCGTGCAGCAAGTTTTNNNNNCCCCAGTAACAAGGTCACAGAGAAGG
BVF12	GCAGTCGTGCAGCAAGTTTTNNNNNAGGGGACAGGATGTAGCTCTCAGG
BVF13	GCAGTCGTGCAGCAAGTTTTNNNNNGGTCATGCAGCCCTCTATTGGT
BVF14	GCAGTCGTGCAGCAAGTTTTNNNNNCAATTTCCGAACACAACCGCCTT
BVF15	GCAGTCGTGCAGCAAGTTTTNNNNNGAGGGGCCAGGATGTAGCTCTCA
BVF16	GCAGTCGTGCAGCAAGTTTTNNNNNAGTCACACAAACCCCAAAGCACCT
BVF17	GCAGTCGTGCAGCAAGTTTTNNNNNCCCAGAGCCCGAGATACAAGGTCA
BVF18	GCAGTCGTGCAGCAAGTTTTNNNNNGCCCAAGACACAAGATCACAGAGACA
BVF19	GCAGTCGTGCAGCAAGTTTTNNNNNTACCCTTTACTGGTACCGGCAGA
BVF20	GCAGTCGTGCAGCAAGTTTTNNNNNATAGAAAAAAGCCAGGCTGTGAC
BVF21	GCAGTCGTGCAGCAAGTTTTNNNNNARGCAYRAGGTGACAGAAATGGGA
BVF22	GCAGTCGTGCAGCAAGTTTTNNNNNCRGCATRAGGTGACAGAGATGGGA
BVF23	GCAGTCGTGCAGCAAGTTTTNNNNNGGCACCTGATCAAAGAAAAGAGGGA
BVF24	GCAGTCGTGCAGCAAGTTTTNNNNNAGTTACTCAGTTCCCCAGCCACA
BVF25	GCAGTCGTGCAGCAAGTTTTNNNNNGAAAAGCCAGTGACCTTGAGTTGT
BVF26	GCAGTCGTGCAGCAAGTTTTNNNNNTCAAAGGGGAAGGACAGAAAGCAA
BVF27	GCAGTCGTGCAGCAAGTTTTNNNNNTCATGTTTATTGGTATCGGCAGCTC
BVF28	GCAGTCGTGCAGCAAGTTTTNNNNNATGGCAGAATCACTCAGTCCCCAA
BVF29	GCAGTCGTGCAGCAAGTTTTNNNNNCGTCTCTCAGTATCCAAGCAGGGTT
BVF30	GCAGTCGTGCAGCAAGTTTTNNNNNACCTAGACTTCTGGTCAAAGCAAACA
BVF31	GCAGTCGTGCAGCAAGTTTTNNNNNGGATTGTACCCCCGAAAAAGGACA
BVF32	GCAGTCGTGCAGCAAGTTTTNNNNNCCCAAGGAATAAGATCGCAAAGACAGG
BVF33	GCAGTCGTGCAGCAAGTTTTNNNNNACCCAAGACACCGTGTTATAGGGA
BVF34	GCAGTCGTGCAGCAAGTTTTNNNNNAAAGAAGTTGACAGTCACTTGTTCTC
BVF35	GCAGTCGTGCAGCAAGTTTTNNNNNATGTGAAAGTAACCCAGAGCTCAAG
BVF36	GCAGTCGTGCAGCAAGTTTTNNNNNGGATGTCTGTCAACGTGGAACCTC
BVF37	GCAGTCGTGCAGCAAGTTTTNNNNNTGTGGAGGGAACATCAAACCCCAA
BJR01	CGAGTCCTGCGGTCTCAAATCNNNNNACTGTGAGTCTGGTGCCTTGTC
BJR02	CGAGTCCTGCGGTCTCAAATCNNNNNACAGTTAACTTGGTCCCTGAACCG
BJR03	CGAGTCCTGCGGTCTCAAATCNNNNNTGAGCCGACTTCCCTCTCCAA
BJR04	CGAGTCCTGCGGTCTCAAATCNNNNNGAGCTGGGTTCCACTGCCAAA
BJR05	CGAGTCCTGCGGTCTCAAATCNNNNNGGACGGAGAGTCGAGTGCCAT
BJR06	CGAGTCCTGCGGTCTCAAATCNNNNNGAGCCTGGTCCCGTTCCCAA
BJR07	CGAGTCCTGCGGTCTCAAATCNNNNNCCGTGTSCCTGGCCCAAAGAA
BJR08	CGAGTCCTGCGGTCTCAAATCNNNNNCACGGTCAGCCTAGAGCCTTC
BJR09	CGAGTCCTGCGGTCTCAAATCNNNNNCGAGCACTGTCAGCCGGGT
BJR10	CGAGTCCTGCGGTCTCAAATCNNNNNCCAGCACTGAGAGCCGGGT
BJR11	CGAGTCCTGCGGTCTCAAATCNNNNNGCGTGCCTGGTCCGAAGT
BJR12	CGAGTCCTGCGGTCTCAAATCNNNNNCCGGCCCCGAAAGTCAGGA
BJR13	CGAGTCCTGCGGTCTCAAATCNNNNNTGGTGCCCGGCCCGAAGT

The raw sequence data were processed and annotated with MiGEC [44] and further analyzed and visualized with VDJtools [45].

### SIV-specific antibody assay

The SIV-specific antibody levels were measured by ELISA using purified SIVmac251 viral lysates as previously described with an acid dissociation step [46, 47]. Ninety-six-well MaxiSorp plates (Nunc, A/S, Denmark) were coated with 100 μl/well of SIVmac251 lysate at 1 μg/ml in 50 mM NaCO_3_ buffer (pH 9.6) and incubated overnight at 4 °C. The plates were washed five times with washing buffer (WB; 0.05% Tween-20 in PBS) and blocked at 37 °C for 2 h with blocking buffer (5% skim milk in WB). After five washes, 40 μl/well of 1 M TRIS (pH 9.5) was added to the plates and then mixed with 100 μl/well of plasma samples that were five-fold diluted with 300 mM acetic acid. The plates were incubated for 3.5 h at RT. After washing five times, anti-monkey IgG-horseradish peroxidase (Sigma Chemical Co., St. Louis, MO) diluted 1:20,000 was added to the plates and incubated for 1 h at 37 °C. The plates were then washed five times and developed with TMB. The reaction was stopped with 2 M sulfuric acid, and plates were spectrophotometrically analyzed at 450 nm.

### SIV-neutralizing antibody assay

Neutralizing antibodies to SIV in plasma were measured in TZM-bl cells (obtained from the NIH AIDS Research and Reference Reagent Program). Eighteen TCID_50_ of SIVmac251 per well were incubated for 1 h at 37 °C with serially diluted heat-inactivated sera and negative control (NC) serum starting with a dilution of 1:20. Ten thousand TZM-bl cells were dispensed to each well together with 20 μg/ml DEAE-dextran. Virus infectivity was determined after 48 h incubation by measuring the luciferase activity induced in TZM-bl cells by Tat protein. Neutralizing activity is expressed as the dilution factor of the serum samples required for 50% (IC50) inhibition of infection (http://www.hiv.lanl.gov/content/nab-reference-strains/html/home.htm).

### Statistical analysis

GraphPad Prism 6 was used for statistical analysis. Survival time was compared using a log-rank test. The AUC was calculated to estimate the cumulative plasma viral load throughout the acute phase of SIV infection. A t-test was used to determine the statistical significance of the differences between groups in the frequency and number of CD4^+^ T cells and their subpopulations and number of IFN-γ-specific SFCs. P-values for the dependence between diversity index and treatment group were computed using ANOVA. *P*-values for analyzing the SIV-specific clone types between groups were computed using ANOVA. The Mann–Whitney test was used to determine the statistical significance of the differences in viral load and the AUC of plasma viral load between groups. The antibody titers were analyzed by two-way ANOVA with Tukey's multiple comparisons test. Values of *P* < 0.05 indicated statistical significance.

## Supplementary Information

**Additional file 1**. **Fig S1**: Cell numbers and ratios of peripheral CD4+ and CD8+ T cells of animals. (A) CD4+ and (B) CD8+ T cells in peripheral blood. (C) CD4+/CD8+ T cell ratio in peripheral blood. (D) Frequency of CD4+CCR5+ cells (among total CD4+ T lymphocytes). The data were from all the animals that survived at the assay time point. Blue and red arrows indicate the times of Pc inoculation and chloroquine treatment, respectively. **Fig S2**: Gating strategies for CD4+ T cell subpopulations. Flow cytometry gating strategies for naïve, effector memory (TEM) and central memory (TCM) CD4+ T cell subpopulations in PBMCs. SSC, side scatter height; FSC, forward scatter height. **Fig S3**: The serum anti-SIV antibody titer for the three SIV-infected groups. **Fig S4**: T-cell repertoire diversity in malaria- and SIV-infected monkeys. (A) SIV infection significantly reduced the repertoire diversity. Change in Chao1 total diversity estimate and Shannon index (entropy of clonotype frequency distributions) with respect to control (0 weeks of SIV infection). (B) Malaria infection increased the Shannon index but not the total repertoire diversity. (C) Increase in the Shannon index was a result of attenuation of dominant clonal expansions. The decrease in the relative frequency of expanded clonotypes occurred at acute malaria infection (week 3 post malaria introduction). **Fig S5**: Malaria-induced changes in repertoire structure and frequency of SIV-specific clonotypes. (A) T-cell repertoires at acute malaria infection were characterized by decreased hydrophobicity (GRAVY index) and increased NDN size, suggesting an increase in polyreactive clonotype frequency. *: P < 0.05, two-tailed paired t-test. (B) SIV-specific clonotypes identified by tetramer sorting (Price et al. data) were characterized by a decreased GRAVY index compared to the pooled repertoire of control samples. P-values were computed using the Kolmogorov-Smirnov test.

## Data Availability

The original contributions presented in the study are included in the article/Supplementary Material. TCRβ sequence data accession number: SAMN23169591-SAMN23169699. Further inquiries can be directed to the corresponding author: XC.

## Abbreviations

SIV: Simian immunodeficiency virus
HIV: Human immunodeficiency virus
AIDS: Acquired immunodeficiency syndrome
SAIDS: Simian acquired immunodeficiency syndrome
Pc: *Plasmodium cynomolgi or P. cynomolgi*
TCR: T cell receptor
AUC: Area under the curve
TEM: Effector memory CD4^+^ T cells
TCM: Central memory CD4^+^ T cells
PBMCs: Peripheral blood mononuclear cells
ELISPOT: Enzyme-linked immunospot assay
IFN-γ: Interferon-γ
DNA: Deoxyribonucleic acid
RNA: Ribonucleic acid
CDR3: Complementarity determining region 3
ART: Antiretroviral therapy
MHC: Major histocompatibility complex
TCID_50_: Tissue culture infectious dose 50 (%)
iRBCs: Infected red blood cells
PCR: Polymerase chain reaction
