# Supplementary material for: Subsequent malaria enhances virus-specific T cell immunity in SIV-infected Chinese rhesus macaques
Source: Cell Commun Signal. 2022 Jul 1;20:101. doi: 10.1186/s12964-022-00910-7 (PMC9248186; doi:10.1186/s12964-022-00910-7)
Supplement: Supplementary file 2 — Additional file 1. Fig S1: Cell numbers and ratios of peripheral CD4+ and CD8+ T cells of animals. (A) CD4+ and (B) CD8+ T cells in peripheral blood. (C) CD4+/CD8+ T cell ratio in peripheral blood. (D) Frequency of CD4+CCR5+ cells (among total CD4+ T lymphocytes). The data were from all the animals that survived at the assay time point. Blue and red arrows indicate the times of Pc inoculation and chloroquine treatment, respectively. Fig S2: Gating strategies for CD4+ T cell subpopulations. Flow cytometry gating strategies for naïve, effector memory (TEM) and central memory (TCM) CD4+ T cell subpopulations in PBMCs. SSC, side scatter height; FSC, forward scatter height. Fig S3: The serum anti-SIV antibody titer for the three SIV-infected groups. Fig S4: T-cell repertoire diversity in malaria- and SIV-infected monkeys. (A) SIV infection significantly reduced the repertoire diversity. Change in Chao1 total diversity estimate and Shannon index (entropy of clonotype frequency distributions) with respect to control (0 weeks of SIV infection). (B) Malaria infection increased the Shannon index but not the total repertoire diversity. (C) Increase in the Shannon index was a result of attenuation of dominant clonal expansions. The decrease in the relative frequency of expanded clonotypes occurred at acute malaria infection (week 3 post malaria introduction). Fig S5: Malaria-induced changes in repertoire structure and frequency of SIV-specific clonotypes. (A) T-cell repertoires at acute malaria infection were characterized by decreased hydrophobicity (GRAVY index) and increased NDN size, suggesting an increase in polyreactive clonotype frequency. *: P < 0.05, two-tailed paired t-test. (B) SIV-specific clonotypes identified by tetramer sorting (Price et al. data) were characterized by a decreased GRAVY index compared to the pooled repertoire of control samples. P-values were computed using the Kolmogorov-Smirnov test. [file 12964_2022_910_MOESM2_ESM.docx]

SUPPLEMENTARY MATERIALS


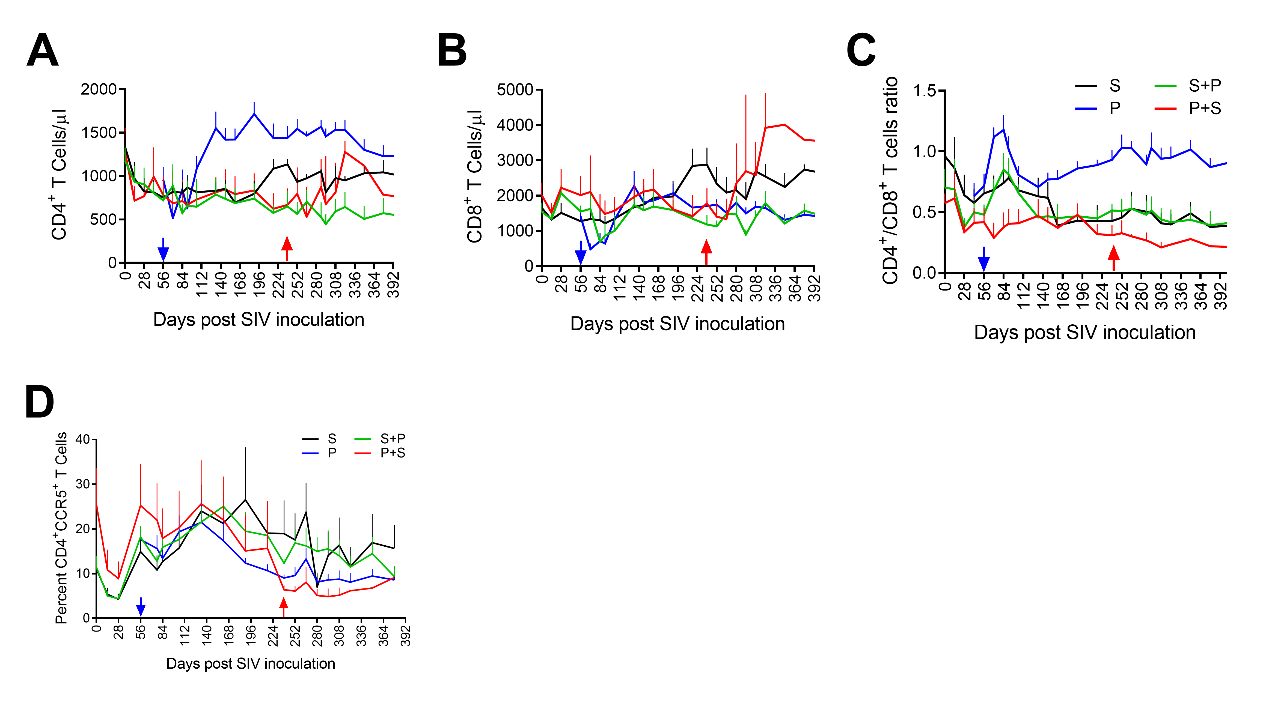


**Supplementary Figure 1. Cell numbers and ratios of peripheral CD4^+^ and CD8^+^ T cells of animals. (A)** CD4^+^ and **(B)** CD8^+^ T cells in peripheral blood. **(C)** CD4^+^/CD8^+^ T cell ratio in peripheral blood. **(D)** Frequency of CD4+CCR5+ cells (among total CD4+ T lymphocytes). The data were from all the animals that survived at the assay time point. Blue and red arrows indicate the times of Pc inoculation and chloroquine treatment, respectively.


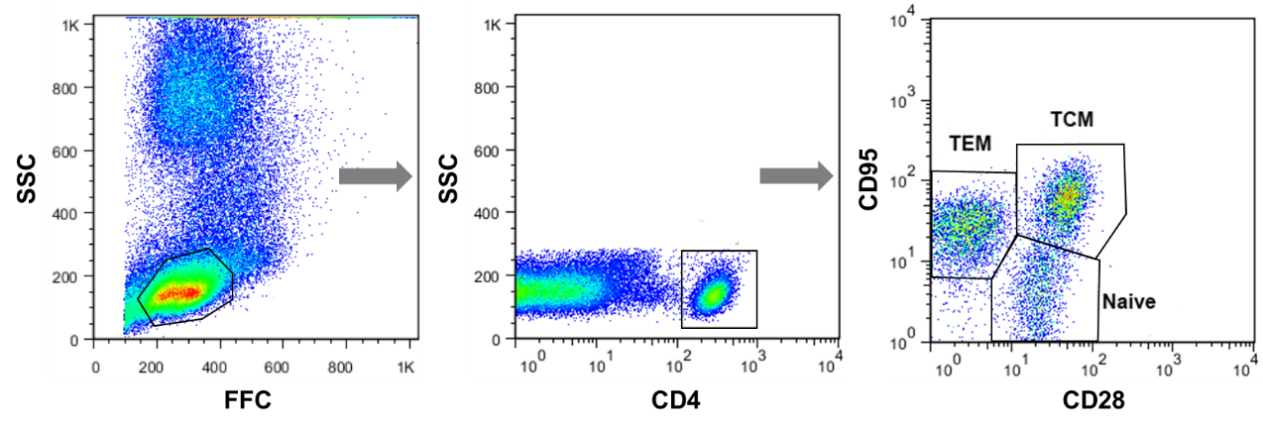


**Supplementary Figure 2. Gating strategies for CD4+ T cell subpopulations.** Flow cytometry gating strategies for naïve, effector memory (TEM) and central memory (TCM) CD4+ T cell subpopulations in PBMCs. SSC, side scatter height; FSC, forward scatter height.


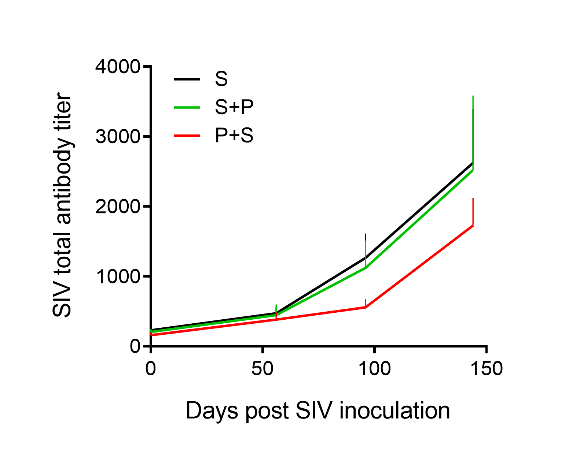


**Supplementary Figure 3**. The serum anti-SIV antibody titer for the three SIV-infected groups.

**
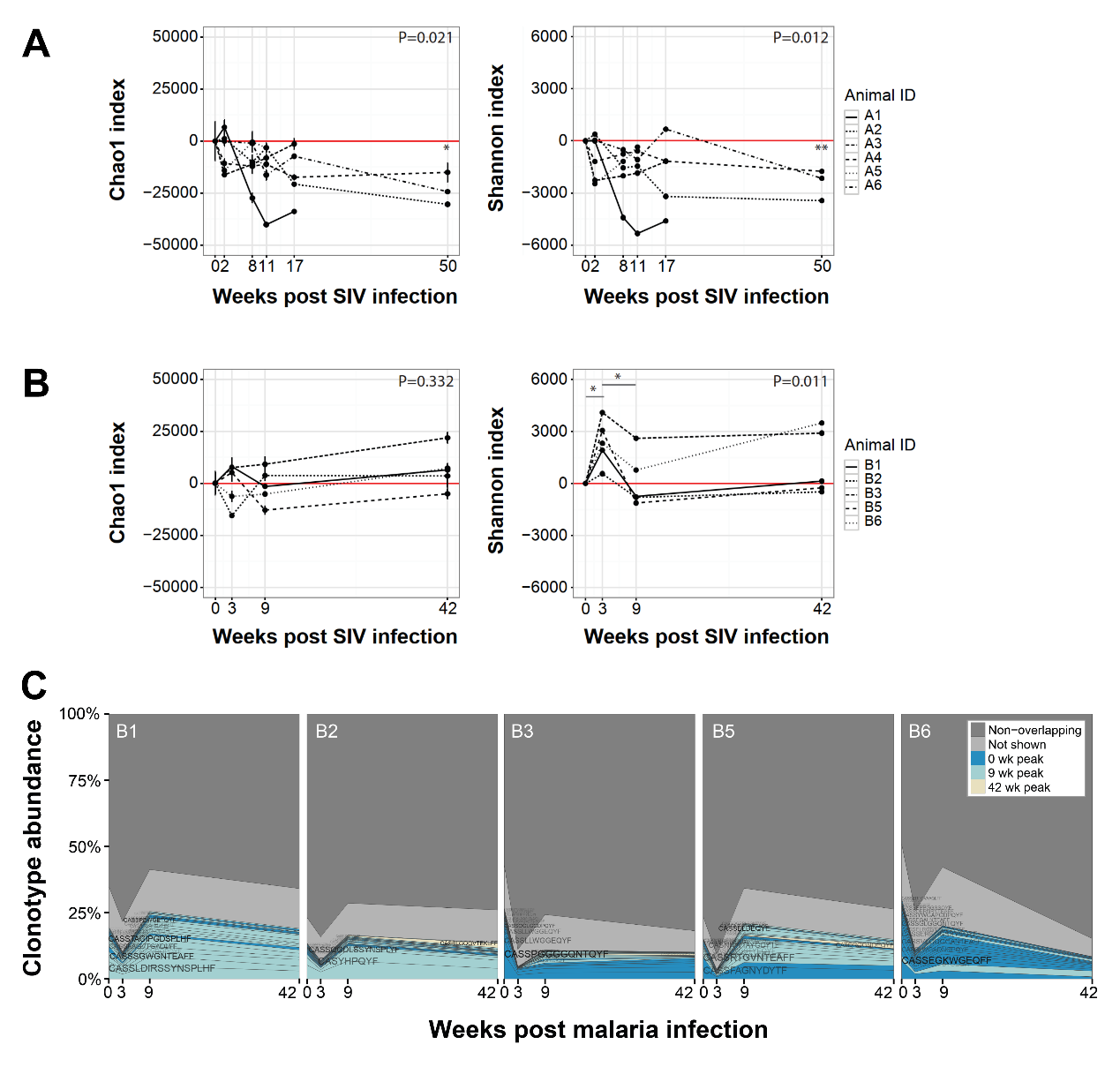
**

**Supplementary Figure 4.** T-cell repertoire diversity in malaria- and SIV-infected monkeys. **(A)** SIV infection significantly reduced the repertoire diversity. Change in Chao1 total diversity estimate and Shannon index (entropy of clonotype frequency distributions) with respect to control (0 weeks of SIV infection). **(B)** Malaria infection increased the Shannon index but not the total repertoire diversity. **(C)** Increase in the Shannon index was a result of attenuation of dominant clonal expansions. The decrease in the relative frequency of expanded clonotypes occurred at acute malaria infection (week 3 post malaria introduction).


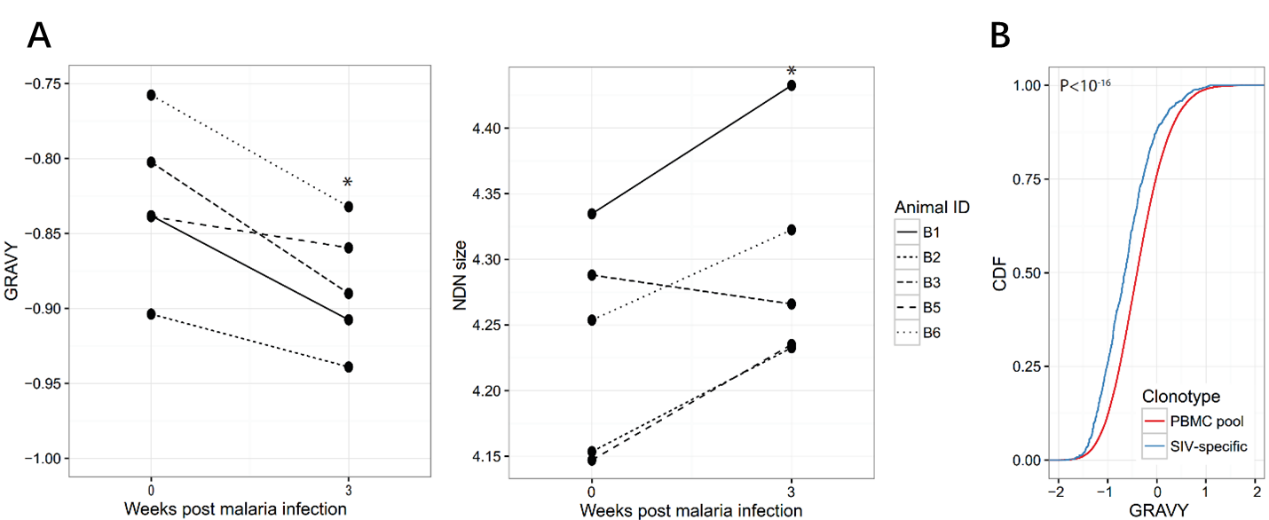


**Supplementary Figure 5.** Malaria-induced changes in repertoire structure and frequency of SIV-specific clonotypes. **(A)** T-cell repertoires at acute malaria infection were characterized by decreased hydrophobicity (GRAVY index) and increased NDN size, suggesting an increase in polyreactive clonotype frequency. *: *P* < 0.05, two-tailed paired t-test. **(B)** SIV-specific clonotypes identified by tetramer sorting (Price et al. data) were characterized by a decreased GRAVY index compared to the pooled repertoire of control samples. *P*-values were computed using the Kolmogorov-Smirnov test.
